# Mr. Semple *on the State of the Bowels in Ulcers*

**Published:** 1813-03

**Authors:** Robert Semple

**Affiliations:** Surgeon. Islington


*To the Editors of the Medical and Physical Journal.*

GENTLEMEN,

FROM a perusal of your Journal, I find many valuable facts. If the following case meet your approbation, you will please to insert it in your next Number. It tends to prove the necessity of minutely attending to the state of the bowels in ulcers.

Benjamin Dockrill, a seaman on-board his Majesty's ship Tigre, had an ulcer on that part of the tibia which is called the shin. I served as a medical officer in that vessel for two or three years, during which time this man's ulcer healed and broke out alternately at very short intervals. Having dressed this ulcer for a long time, I was induced to pay attention to the case. Every kind of application was tried to the sore without any effect. One time, in particular, I recollect dressing this sore, in a heavy storm, in the Gulf of Lyons, when I observed that the part looked livid, and its surface covered with a black, grumous, and fetid matter. I said to the man, What have you been doing to your leg this morning? (This is a very common question at sea, as the sailors who want to trick the doctor frequently bind a halfpenny on their sores, and thereby produce a very foul-looking ulcer.)—The man said, Sir, I have done nothing to my my leg: and moreover he replied, that he had an ardent desire to return to his duty. I then asked him, in what state his bowels were. He said that he had been running to the *head** almost every minute, and passed much blood, accompanied with urgent tenesmus. His tongue was foul, and he had no appetite. I immediately gave him some calomel, I think four or five grains. Next morning he told me he had passed a good night, the tenesmus had nearly gone off, and the sore looked clean and healthy. A grain of calomel was given to him every other night for some time, and the sore got well. The ulcer, however, frequently afterwards broke out, from the ship's ropes, &c. rubbing off the thin skin, although he was ordered to wear a fender on the part where the sore had been. I invariably found, however, that, whenever the sore put on an healthy appearance, this man's bowels were uniformly disordered. I am, Gentlemen,

*Islington*, Your obedient humble Servant,

*Jan.* 12, 1813. ROBERT SEMPLE, Surgeon.

