# Corrigendum

**Published:** 1813-04

**Authors:** 


### *CORRIGENDUM.*

*In our last Number, page* 201, *line* 15, *for* "healthy," *read* "unhealthy,"

